# Schizophrenia and reelin: a model based on prenatal stress to study epigenetics, brain development and behavior

**DOI:** 10.1186/s40659-016-0076-5

**Published:** 2016-03-11

**Authors:** Ignacio Negrón-Oyarzo, Ariel Lara-Vásquez, Ismael Palacios-García, Pablo Fuentealba, Francisco Aboitiz

**Affiliations:** Departamento de Psiquiatría, Escuela de Medicina, and Centro Interdisciplinario de Neurociencia, Pontificia Universidad Católica de Chile, Santiago, Chile

**Keywords:** Schizophrenia, Prefrontal cortex, Prenatal stress, Functional connectivity, DNA methylation, *Reelin*

## Abstract

Schizophrenia is a severe psychiatric disorder that results in a significant disability for the patient. The disorder is characterized by impairment of the adaptive orchestration of actions, a cognitive function that is mainly dependent on the prefrontal cortex. This behavioral deficit, together with cellular and neurophysiological alterations in the prefrontal cortex, as well as reduced density of GABAergic cells and aberrant oscillatory activity, all indicate structural and functional deficits of the prefrontal cortex in schizophrenia. Among the several risk factors for the development of schizophrenia, stress during the prenatal period has been identified as crucial. Thus, it is proposed that prenatal stress induces neurodevelopmental alterations in the prefrontal cortex that are expressed as cognitive impairment observed in schizophrenia. However, the precise mechanisms that link prenatal stress with the impairment of prefrontal cortex function is largely unknown. Reelin is an extracellular matrix protein involved in the development of cortical neural connectivity at embryonic stages, and in synaptic plasticity at postnatal stages. Interestingly, down-regulation of reelin expression has been associated with epigenetic changes in the *reelin* gene of the prefrontal cortex of schizophrenic patients. We recently showed that, similar to schizophrenic patients, prenatal stress induces down-expression of reelin associated with the methylation of its promoter in the rodent prefrontal cortex. These alterations were paralleled with altered prefrontal cortex functional connectivity and impairment in prefrontal cortex-dependent behavioral tasks. Therefore, considering molecular, cellular, physiological and behavioral evidence, we propose a unifying framework that links prenatal stress and prefrontal malfunction through epigenetic alterations of the *reelin* gene.

## Background

Schizophrenia is a chronic psychiatric disorder that affects 0.5–1 % of the world’s population. It is characterized by a complex set of disturbances of thought, perception, and affective and social behavior that result in high social disability [1]. Although the causes of this disorder are not completely understood, clinical research has identified some factors that provide insight into the pathophysiology of this disease [2]. For example, schizophrenia is characterized by impairment of cognitive functions dependent on the prefrontal cortex (PFC; [3]), which coincides with cellular and neurophysiological alterations observed in the PFC of schizophrenic patients [4, 5]. It is also known that prenatal stress (PNS) is an important etiologic factor for the development of this disorder [6], which implies that PNS induces neurodevelopmental alterations in the PFC that are manifested as cognitive alterations observed in schizophrenic patients. In this review, we propose that PNS-induced epigenetic changes in the *reelin* gene, which codes for an extracellular protein involved in cortical development, could be a molecular link between prenatal stress and PFC dysfunction.

## The deficit in cognitive control in schizophrenia suggests functional impairment of PFC function

The symptomatology of schizophrenia has provided some clues about the neurophysiology of the disorder. Symptoms are classified as cognitive, positive, and negative [1]. Among these symptoms, cognitive impairments are especially relevant because they impact on the normal life performance of patients. These cognitive impairments, like reduced working memory [3, 7–9], selective attention [10], and set-shifting [11], can be globally grouped as a detriment to executive control: i.e. the proper orchestration of thoughts and actions in accordance with internal goals [12]. It has been suggested that the degree of cognitive impairments, and not the severity of psychosis, is the best predictor of long-term functional outcome for affected individuals, leading to the view that cognitive deficits are the core abnormalities of the illness [13, 14]. Thus, the deficit of executive control appears to be a hallmark of schizophrenia [3, 9, 15].

The PFC is considered the main brain area involved in executive control [12, 16]. The cognitive symptoms of schizophrenia suggest a functional impairment in the PFC as a core neurological dimension, a feature known as “hypofrontality” [3]. This functional deficit seems to be strongly related to altered neural oscillatory synchrony in the PFC [17–19], functional alterations that correlate with cognitive deficits in schizophrenic patients [4, 20]. The gamma-frequency band (30–80 Hz), the most evident neurophysiological parameter affected in schizophrenia, is required for the implementation of executive control by the PFC [21, 22], suggesting that altered gamma oscillations are implicated in cognitive dysfunction [23]. It has been shown that transmagnetic stimulation applied to the gamma-frequency band in the PFC alleviates cognitive symptoms in some schizophrenic patients [24].

The PFC of schizophrenic patients also displays profound alterations at the cellular level, like a reduction of the mean clustering distance between cells by alterations in neuropile volume [25]. It has also been observed that schizophrenics have fewer dendritic spines in pyramidal neurons than non-schizophrenic post-mortem subjects [26]. However, the inhibitory GABAergic neurons seem to be the most affected neuronal population in the PFC of schizophrenic patients. One of the most consistent findings in postmortem studies in the PFC of individuals with schizophrenia is the reduced mRNA expression of GAD67, the enzyme that synthesizes GABA [27]. In addition, reduced density of GABAergic cells, and decreased amounts of inhibitory axon terminals have been found post-mortem in the PFC of schizophrenic patients [5, 28, 29]. This evidence has led to consider schizophrenia as a disease of impaired inhibitory transmission in the PFC [30–32]. Given that GABAergic interneurons are strongly implicated in the emergence of gamma-frequency oscillations in cortical networks [33–35], this evidence suggests that cellular impairments may underlie neurophysiological PFC alterations related to cognitive impairment in schizophrenia [32].

## The effects of prenatal stress on the PFC as a neurodevelopmental factor for schizophrenia

Some cognitive and neurophysiological alterations observed in schizophrenic patients are evident during early childhood, before patients manifest diagnosed symptoms [36–39]. This, together with the prenatal development of cellular components altered in schizophrenia, like cortical microcircuit connectivity and GABAergic transmission [26, 40, 41], all suggest that schizophrenia can also be considered a neurodevelopmental disorder, especially focused on the development of the PFC [38, 42, 43]. Thus, current evidence indicates that neurodevelopmental cellular alterations in the PFC, particularly those related to inhibitory transmission, is associated to abnormal functional connectivity in the PFC, resulting in an impairment of executive functions in schizophrenic patients [43]. But, how are these neurodevelopmental alterations in the PFC acquired?

Among the several acquired and environmental factors involved in the development of schizophrenia [44], the suffering of threatening situations by the pregnant mother during gestation, i.e. PNS, has been considered a strong environmental risk factor [6]. In support of this idea, it has been shown that the number of individuals with diagnoses of schizophrenia is significantly higher among individuals with prenatal loss of their fathers than among individuals whose fathers died during their first year of childhood [45]. Accordingly, van Os and Selten [46] found a higher cumulative incidence of schizophrenia among individuals prenatally exposed to the 1940 invasion of the Netherlands by the German army, suggesting that maternal stress during pregnancy may contribute to the development of vulnerability to schizophrenia. Similarly, Betts et al. [47] showed that stressful prenatal life events predicted psychotic experiences in adulthood. Finally, Levine et al. [48] found that PNS associated to exposure to the holocaust constitutes a consistent risk factor for schizophrenia. Thus, taking in consideration the essential role of PNS as a development risk factor for schizophrenia, and that this disorder is characterized by functional impairment of the PFC, two critical questions arise: (1) Does PNS produce functional impairment of the PFC associated with schizophrenia? And if so, (2) How does this process occur?

**Fig. 1:**
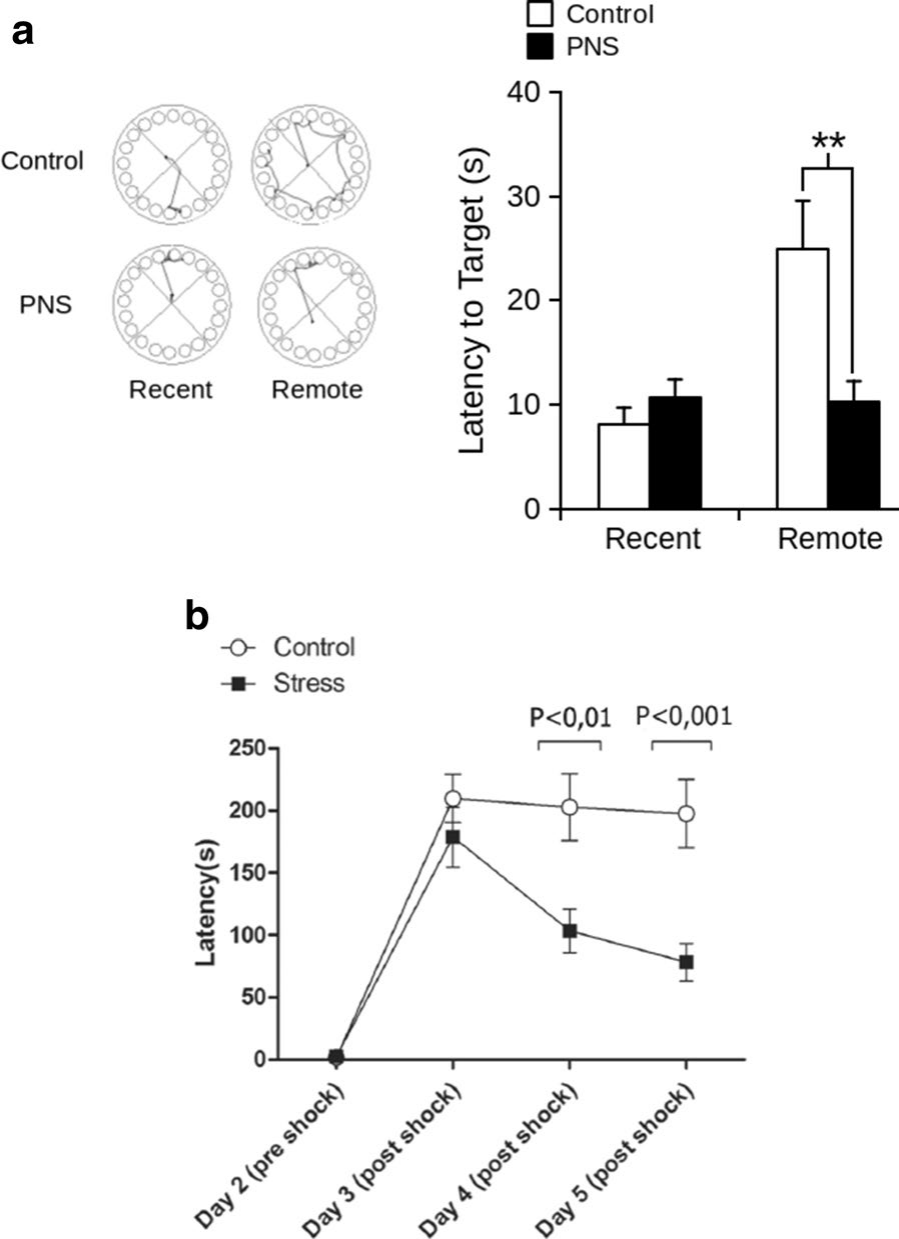
Prenatal stress produces long-term persistence of spatial memory and decreases learning retention in a passive avoidance test. **a** Control and PNS mice were trained for 4 days to locate the escape in the Barnes maze test. Latency to find the escape was assessed 1 (recent memory) and 10 days (remote memory) after training. *Right panel* example of tracking plots for 2 mice (Control and PNS) in the Barnes maze during recent and remote memory testing. *Left panel* *bar chart* showing the latency to escape for both groups of mice in the two memory conditions (*P < 0.05; Bonferroni post hoc after 2-way ANOVA). **b** In the passive avoidance learning-retention test, latency time to enter the dark chamber of the shuttle box was measured, where a mild foot shock was delivered on day 2. There were significant differences (Bonferroni post hoc after 2-way ANOVA) in the latency time between control and PNS rats on days four and five post-shock. Data are presented as mean ± SEM. Adapted from [55, 125]

**Fig. 2:**
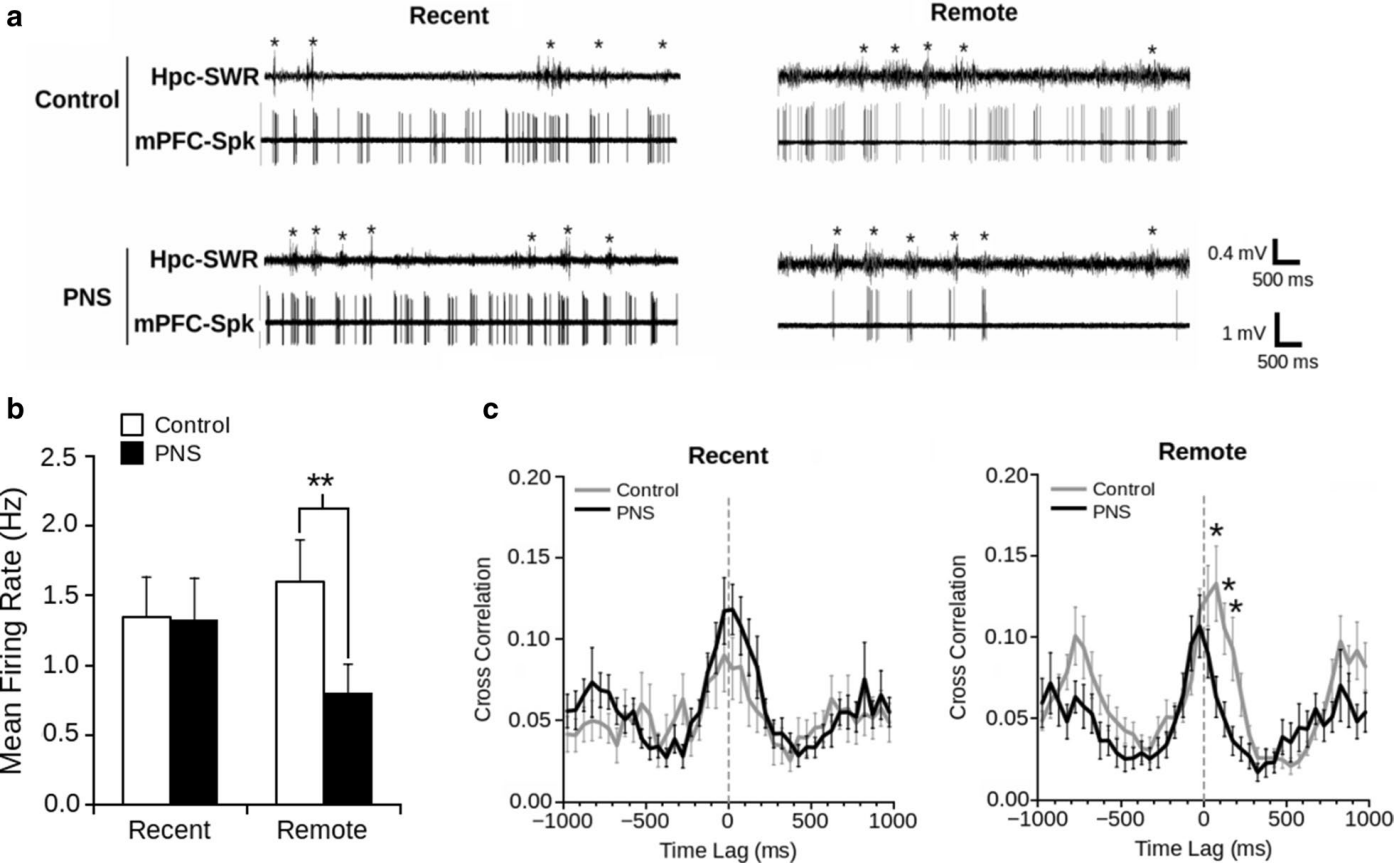
Prenatal stress decreases firing rate in the PFC and disrupts functional connectivity between the PFC and hippocampus. Control and PNS mice were subjected to in vivo local field potential recording under urethane anesthesia in the hippocampus and PFC, after either recent or remote memory testing in the Barnes maze. **a** Representative recordings for each group and condition displaying the hippocampal LFP filtered at 100–300 Hz (*upper*) and its correlative prefrontal LFP filtered at 300–5 Hz (*lower*). *Asterisks* indicate sharp wave ripples (SWR) cross-correlated with spikes from PFC cells. **b** Mean firing rate of spontaneously firing neurons in the PFC (**P < 0.01; Mann–Whitney U test). Data are shown as mean ± SEM. **c** Mean normalized cross-correlation between significantly correlated PFC single units to hippocampal SWRs. Note the significant difference of discharge in the PFC 200 ms after the ripple onset in remote memory in PNS group (*P < 0.05; Wilcoxon signed rank test). Data are presented as mean ± SEM. Adapted from [55]

It has been shown in humans that stressing situations experienced by the mother during pregnancy affect PFC-dependent cognitive functions of the offspring, like working memory, control of anxiety, and learning strategies [49–52]. Similarly, research in rodents have shown that PNS affects cognitive functions dependent on the limbic and prelimbic cortex, (the rodent homologue and analogue to the human PFC [53]), manifested as impairment of working memory [54], increase of aversive remote memory [55] (Fig. 1) or decreased recall of the extinction of conditioned fear [56]. These data indicate that PNS affects cognitive functions dependent on the PFC at adulthood [57, 58], which could be related to the pathogenesis of schizophrenia [48, 59]. At a neurophysiological level, PNS alters neuronal synchronization between the PFC and the hippocampus, connectivity relevant to the consolidation of memories [58, 60] together with a decreased firing rate in the PFC in vivo [55] (Fig. 2). Coincidently, these neurophysiological alterations are paralleled with the persistence of aversive remote memory [53, 55] (Fig. 1), a PFC-dependent cognitive function [61].

At the cellular level, there is abundant evidence suggesting that PNS affects the correct development of the PFC in rodents. For example, dendritic ramification of pyramidal neurons is disrupted in prenatally stressed adults rats [62], morphological alterations that are also evident during earlier developing stages like early childhood [63] and adolescence [64]. PNS not only affects pyramidal neurons in the PFC, but also the development of inhibitory neurons. For example, PNS decreases the number of PV+ interneurons in the PFC [65], and delays tangential migration of inhibitory neurons in the developing neocortex [64]. This is especially important since, as mentioned above, a reduction in inhibitory neuronal activity in the PFC has been proposed as an important physiopathological feature of schizophrenic patients [31, 32]. Altogether, these data suggest that PNS induces cellular neurodevelopmental alterations expressed as neurophysiological alterations in the PFC, as observed in schizophrenia [66]. However, the precise molecular mechanism by which PNS contributes to the development of schizophrenia remains elusive.

## Reelin as a molecular candidate for cellular alterations in schizophrenia

Among molecular candidates involved in the development of schizophrenia [66–69], reelin seems to be an important link between prenatal stress and cellular and physiological alterations observed in schizophrenia. Reelin is a 400~ kD extracellular matrix glycoprotein coded by a 450-bp gene located in the human chromosome 7q22 and in the murine chromosome 5 [70]. The *reelin* gene has multiple cis elements, including for transcription factors involved in neurodevelopment like Sp1, Tbr-1 and Pax6, and for signal transduction like CREB [71, 72]. The protein exerts its function through the union with the VLDLR and ApoER2 receptors. This coupling elicits the intracellular phosphorylation and activation of the adaptor protein disabled 1 (mDab1), which initiates a signaling pathway that ends with the modulation of the cytoskeleton of actin and microtubules [73]. Among the several molecular candidates for the physiopathology of schizophrenia (for review see [74]), clinical and preclinical evidence indicates reelin is a relevant component [75–78]. Below we review the evidence that supports reelin as a molecular candidate for the cellular disruptions produced in schizophrenia.

### Reelin participates in prenatal development and shapes post-natal neural connectivity in the neocortex

Reelin protein is expressed in mammals during brain development, principally by Cajal-Retzius neurons in superficial layers of the neocortex and the hippocampus [79–81]. In rodents, cortical and hippocampal Cajal-Retzius neurons degenerate progressively to postnatal day 14 [82, 83], limiting the production and secretion of reelin to GABAergic interneurons from postnatal day 8 to adulthood [83–85]. The role of reelin in neurodevelopment has been well demonstrated, especially by regulating the radial migration of excitatory neurons and the establishment of the “inside-out” neurogenetic gradient [73, 86–88]. The *reeler* mouse (homocygote knock-out for *reelin*, and thereby deficient for reelin; [89]), has a clear disruption of cortical layers. In addition it has been demonstrated that *reeler* mice display an aberrant disposition of inteneurons in the neocortex [90, 91], and that positioned neurons fail to connect to each other and to form a correct cortical architecture [73, 80, 92]. On the other hand, the heterozygous *reeler* mouse (HRM), which has 50 % expression of reelin and is used as a model for schizophrenia [93], does not have the inversion of the cortical layers observed in homozygous *reeler* mice [94]. However, it has reduced dendritic length and complexity and spine density compared with wild type animals [95, 96]. Importantly, the HRM mouse also displays decreased cortical GABA biosynthesis [97] and decreased cortical GAD67 [96, 98].

Reelin also participates in the remodeling of neuronal connectivity in the adult brain modulating synaptogenesis [99], synaptic plasticity [100–104] and neurotransmitter release [105]. The HRM display a decrease in spine density in parallel to lack of NMDA receptor dependent long-term potentiation in the PFC [106]. Furthermore, in vivo enhancement of reelin signaling increases cognitive ability, synaptic plasticity, and dendritic spine density [103]. Altogether, this evidence indicates that reelin modulates cortical neuronal connectivity in both pre- and postnatal stages.

### Reduced expression of reelin and hypermethylation of the reelin promoter is found in the PFC of schizophrenic patients

Impagnatiello et al. [107] were the first to report that *reelin* mRNA and protein expression were significantly lower in the PFC of post-mortem schizophrenic patients. This reduction in reelin expression reached 50 %, and was especially evident in superficial cortical layers [107]. This finding was later replicated by others [76, 108–110].

In recent years it has been proposed that epigenetic mechanisms like DNA methylation play an important role in the gene-environment interaction in the development of psychiatric disorders, including schizophrenia [111–113]. It has been shown that the promoter of the *reelin* gene, together with sequences flanking exon 1, contains near 120 CpG islands [114]. The *reelin* promoter in in vitro assays is methylated in non-reelin expressing cells, and demethylated in reelin expressing cells [114], indicates that *reelin* expression is controlled by the methylation of its promoter. The *reelin* promoter is hypermethylated in the brain of schizophrenic post-mortem patients [39, 72, 115–117]. This reduction of *reelin* and hypermethylation of its promoter in schizophrenic patients is restricted to GABAergic neurons in the PFC [118]. Thus, the down-regulation of *reelin* expression documented in schizophrenic patients might be the consequence of inappropriate promoter hypermethylation [114], especially in GABAergic neurons.

### Reduced expression of reelin in animal models produces schizophrenic-like features

Genetic animal models in which the expression of *reelin* is decreased display cognitive, physiological and cellular features similar to those found in schizophrenic patients. For example, *reeler* mice show increased cognitive impairment and stereotypic behavior [98]. Importantly, the HRM displays a deficit in PFC-dependent cognitive abilities, such as reversal learning and recall of fear extinction [106, 119], together with impairment in the acquisition of operant tasks [120] and increased anxiety [121]. Moreover, overexpression of *reelin* prevents the manifestation of behavioral phenotypes related to schizophrenia [122]. Although it has not been as heavily described as the *reeler* mice, the HRM also displays cellular features in the PFC similar to those of schizophrenic patients, such as decreased GAD67 mRNA, GAD67 protein, and fewer GAD67 positive cells in the PFC [96, 119]. Finally, *reelin* knockdown animals specifically in the PFC show decreased working memory [123]. Together, this evidence suggests a critical role for reelin in the deficits observed in schizophrenia.

## Interaction between PNS, reelin expression and PFC-cognitive impairment observed in schizophrenia

**Fig. 3:**
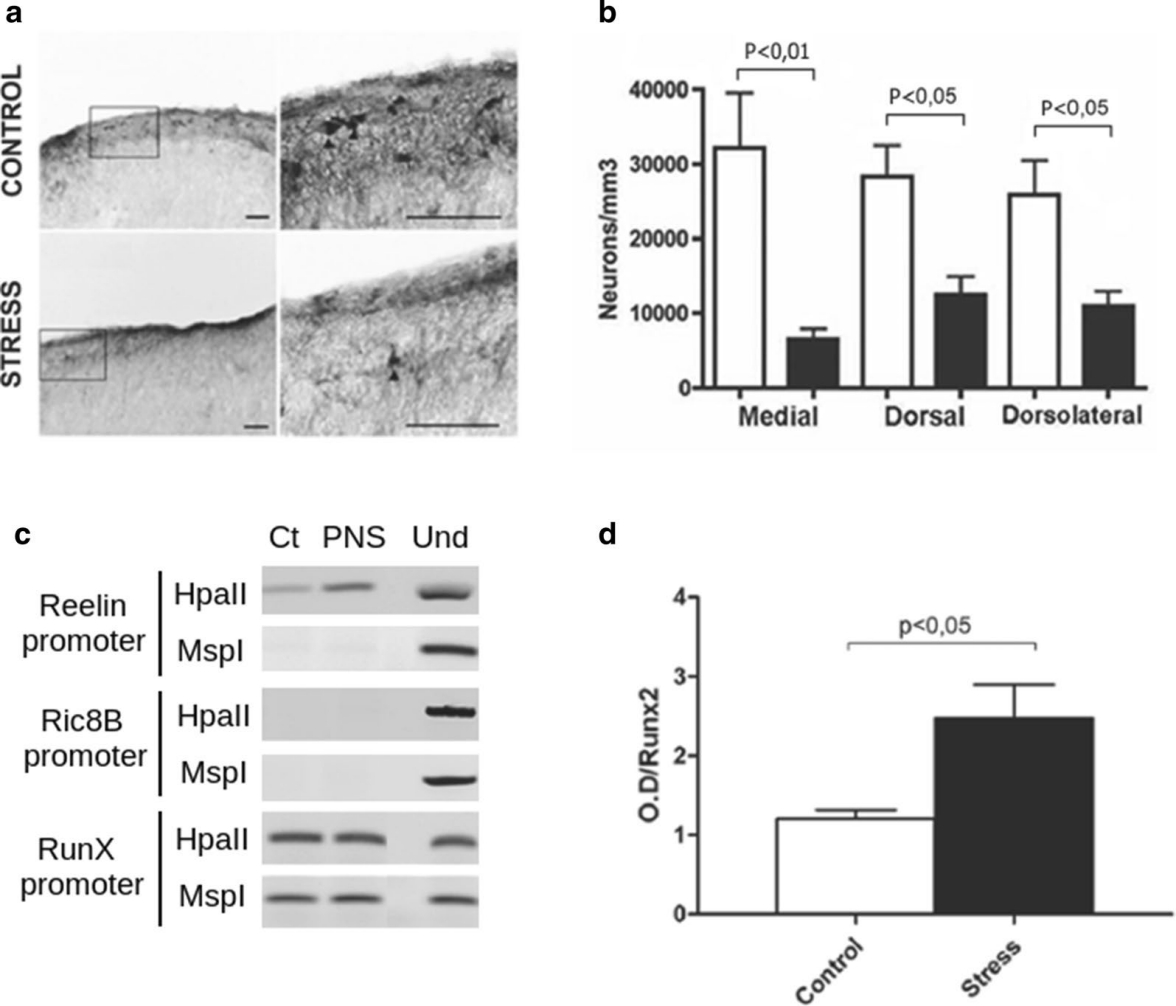
Prenatal stress reduces *reelin* expressing neurons and increases *reelin* methylation in the PFC. **a** Microphotographs of reelin-expressing neurons in the PFC prenatally (E20). Control brains show numerous clusters of Cajal-Retzius neurons while the PNS (*stress*) group shows only a few isolated Cajal-Retzius neurons. *Scale bars* 50 μm. **b** *Bar chart* of neurons immunoreactive for reelin (expressed as neurons/mm^3^). Values are mean ± SEM. **c** Representative agarose gel electrophoresis showing PCR product of the amplification of the distal *reelin* promoter region containing an HpaII site (−786/−625). As control, PCR product of the amplification of *Ric8B* promoter (digestion insensitive to methylation) and *RunX* promoter (without HpaII sensitive regions) after digestion with HpaII or MspI. **d** DNA methylation differences between control and PNS (*stress*) groups were quantified by determining changes in pixel density at the bands amplified by PCR and visualized through conventional DNA electrophoresis. Adapted with modifications from [125]

Prenatal stress may induce DNA methylation of several gene promoters, including *reelin* [124]. Our research and that of others have shown that PNS in rodents reduces the expression of *reelin* in the PFC in adulthood [125, 126] (Fig. 3), which is accompanied by increased methylation of the *reelin* promoter [125, 126] (Fig. 3). PNS-induced own-regulation of reelin by DNA methylation is similar to that found in schizophrenic patients [115]. Together, this evidence places *reelin* and the epigenetic regulation of its expression as likely targets for the development of PNS-induced neuropsychiatric pathology. We have shown that PNS impairs cognitive functions dependent on the PFC, such as the consolidation of memory and passive avoidance (Fig. 1; [55, 125]). In the first case, this behavioral impairment is paralleled with decreased neural activity in the PFC and altered neuronal synchrony between the PFC and hippocampus [55] (Fig. 2). Altogether, the evidence suggests a relationship between epigenetic alterations induced by PNS on the *reelin* gene, with PFC impairment observed in schizophrenia.

## Conclusion

**Fig. 4:**
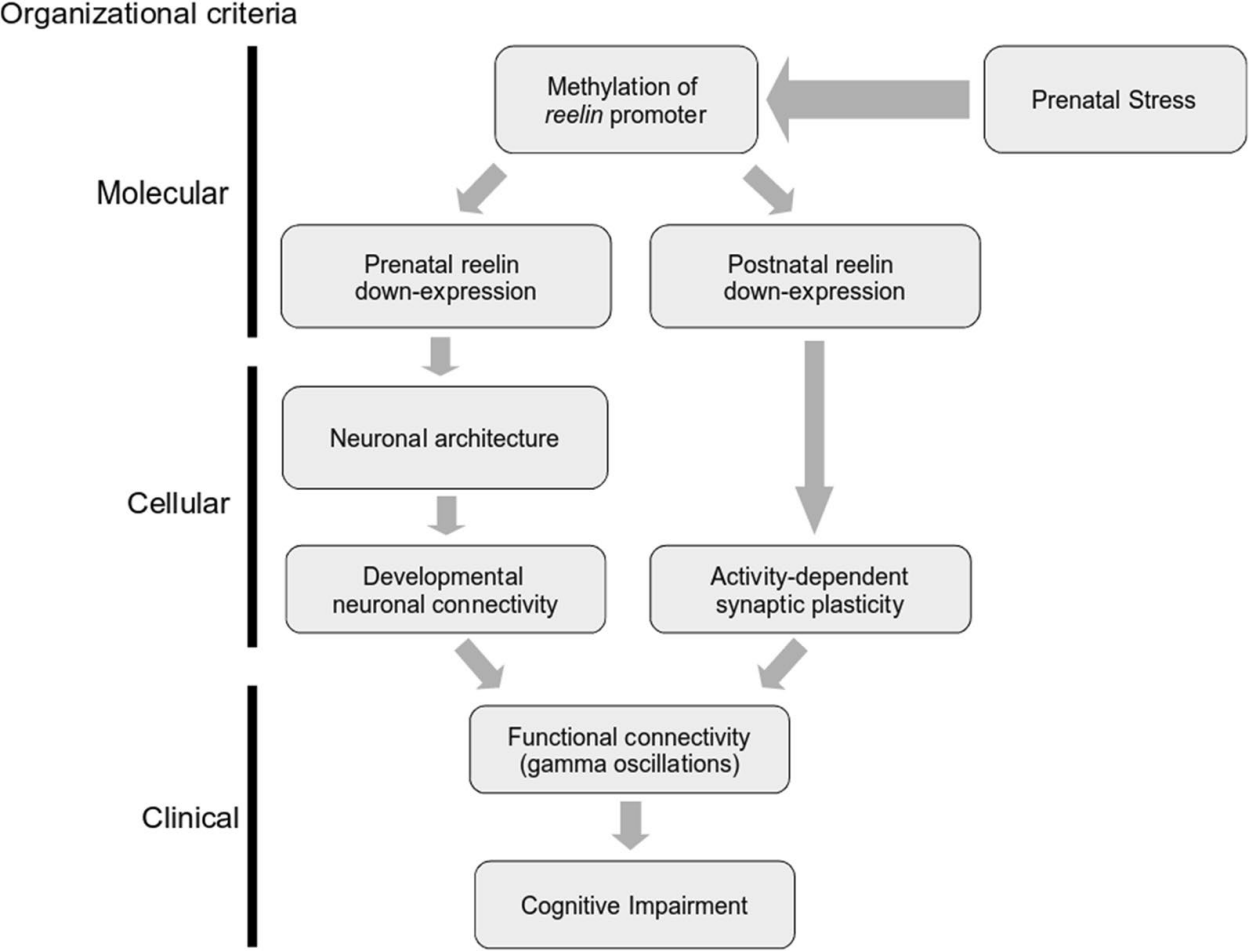
Theoretical model to link prenatal stress, reelin and PFC cognitive impairment. This model, which unifies molecular, cellular and clinical organizational criteria, proposes that PNS induces methylation of the *reelin* promoter, resulting in the down-expression of *reelin* in synthesizing cortical neurons, the effects of which begin to manifest themselves in the prenatal development and are maintained during consequent developmental stages to adulthood. In prenatal stages, down-expression of *reelin* produces alterations in neuronal cytoskeleton dynamics that result in deviances from normal neuronal architecture of the PFC, such as altered positioning of neurons, reduction of dendritic complexity, and reduction of the number of GABAergic neurons, resulting in altered developmental neuronal connectivity. Due to the stability of epigenetic alterations, down-expression of *reelin* continues during postnatal stages to adulthood, where it is manifested as an impairment of activity-dependent synaptic plasticity. These structural and functional alterations modify neuronal connectivity, especially in GABAergic interneurons, leading to altered functional connectivity in the PFC expressed as decreased oscillatory activity, especially at the gamma-frequency band. Given that functional connectivity is required for the implementation of executive function by the PFC, these changes can manifest themselves as cognitive and behavioral impairments dependent on the PFC. Finally, this model does not exclude other candidate genes that may also be affected by PNS and impact on the symptomatology of schizophrenia

Considering molecular, histological, and physiological evidence based on the PNS paradigm, we propose a model that links molecular, neurophysiological and cognitive alterations observed in schizophrenia (Fig. 4). In this model, PNS-induced epigenetic modifications in the *reelin* promoter produce down-expression of *reelin* during prenatal development [125, 126]. As several other researchers have shown, this results in the prenatal reduction of the number of interneurons synthesizing GABA, together with an aberrant layer positioning of cortical interneurons [31, 91, 127] and the reduction of dendritic length and complexity of pyramidal neurons in the PFC [63, 95, 96]. Thus, PNS may impair the development of correct neuronal connectivity in the PFC before birth, which in subsequent developmental stages is expressed as an aberrant functional connectivity of the neural network in the PFC, or between PFC and other structures [19, 55]. Finally, the alteration of the functional connectivity required to implement executive control by the PFC [21, 22] is evidenced as abnormal PFC-dependent cognitive functions [4, 20, 23], which are a hallmark of schizophrenia [3, 9, 19].

Note however that this model does not imply that *reelin* is the only link between PNS and schizophrenia, as other PNS-regulated genes like GAD67 and BDNF [126, 128] may also impact on the symptomatology of schizophrenia. Finally, due to lack of experimental evidence, this model has some gaps in important aspects. For example, it is unknown whether PNS affects the neurophysiological properties of GABAergic interneurons and therefore, the proper functioning of the prefrontal neural network. It is also unknown how these cellular alterations induced by PNS affect the functional connectivity within the PFC and between the PFC and other structures, specifically during the implementation of executive behavioral functions. Future research will assess these undetermined issues, which can contribute to understanding the neurobiology of schizophrenia.

## Abbreviations

PNS: prenatal stress
PFC: prefrontal cortex
HRM: heterozygous *reeler* mice
